# Call for joint informed consent in athletes with inherited cardiac conditions

**DOI:** 10.1136/openhrt-2016-000516

**Published:** 2017-01-30

**Authors:** Rui Providencia, Carina Teixeira, Oliver R Segal, Augustus Ullstein, Pier D Lambiase

**Affiliations:** 1 Barts Health NHS Trust, London, UK; 2 Centre for Psychiatric Rehabilitation, Boston University, Boston, Massachusetts, USA; 3 39 Essex Chambers, London, UK; 4 University College of London, London, UK

**Keywords:** sports, athletes

## Abstract

Informed consent is of the utmost importance, especially in complex clinical situations where patients may be exposed to a life-threatening risk. A particularly complex example is the eligibility of competitive athletes with inherited cardiac conditions on medical grounds, especially when the risk is low or unquantifiable. The rationale and benefits of a joint informed consent for athletes to compete with potentially life-threatening cardiac conditions are discussed in this manuscript.

**Key messages**

**What is already known about this subject?**

- Obtaining an informed consent in athletes with arrhythmic disorders has been mentioned for a few years, but so far it has not been incorporated into guidelines, or extensively discussed in the literature.

**What does this study add?**

- This is the first paper to focus on this concept, providing a thorough rationale for its use, and highlighting all the advantages of implementing informed consenting of athletes, both for the athlete and for the physician.

**How might this impact on clinical practice?**

- Obtaining an informed consent for all athletes, or athletes with potential arrhythmic disorders, will improve transparency, doctor-patient communication, knowledge of athletes and sports teams about cardiac conditions, improve availability of staff trained in cardiopulmonary resuscitation and automatic external defibrillators, and ultimately it will allow empowerment of patients.

## Background

The diagnosis of a heart rhythm disorder or cardiomyopathy in a competitive athlete may lead to dilemma and challenging choices, associated with medical, ethical and legal controversy.^1^ Very often, the degree and extent of sudden cardiac death risk exposure in a specific individual is impossible to quantify. Not only can individuals who qualify to participate in sport after thorough cardiac screening and still experience ventricular fibrillation,^2^ but conversely those testing positive for cardiac conditions, like hypertrophic cardiomyopathy, may live long and healthy lives without serious arrhythmic events.^3^

Obtaining patients' informed consent has now become a routine and mandatory part of medical practice (‘Practising medicine without consent may constitute assault, actionable without proof of physical damage’.).^4^ ^5^ We believe the same should apply to the setting of preparticipation screening and eligibility decisions. However, this situation is unique and complex because of the different parties involved (athlete/patient, physician(s) *and* sports team/organisation), the challenges of predicting risk in individuals with complex cardiac conditions competing under extreme physical stress and that disqualification on medical grounds may have devastating psychological and financial implications to the individual. This paper will focus on cardiac disorders alone. Concussions or musculoskeletal injuries are beyond this remit.

### From the first proposal into the currently suggested framework

The need for an athlete informed consent model was proposed several years ago.^6^ The concept was based on a ‘strong libertarian philosophy that would enable a physically impaired athlete to voluntarily assume the risk of a potentially serious injury, which is not medically certain, or even likely, to occur’.^6^ However, this notion has not made its way into the routine medical decision-making process. British law contemplates the concept of *volenti non fit injuria* (from Latin: *to a willing person, harm is not done*)^7^ Applied to the athlete with a heart rhythm disorder, this would mean that if the athlete is willing to undergo voluntarily exposure to risk, and fully knows and understands the extent of the possible consequences, there is no one to blame in case something happens.

Sometimes, athletes and families are willing to sign waivers preventing future legal claims against physicians, or sports clubs, in the case of an adverse event.^8^ ^9^ Legal issues may also arise in patients who have previously undergone cardiac screening which failed to detect a cardiac condition but ultimately experience fatal events. Therefore, we believe the signing of a joint informed consent form by all athletes, and not only those with ‘grey zone’ cardiac conditions (examples presented in box 1)^10^ ^11^ should be mandatory, as all athletes, no matter how thorough their screening, may potentially be at risk of sudden cardiac death and should be made aware of that. This is particularly relevant in the context of non-diagnostic findings, for example, subtle ECG or imaging anomalies identified on investigation of cardiac symptoms, family screening for sudden arrhythmic death syndrome or preparticipation screening. Even though preparticipation screening is controversial and has recently been called into question by an Israeli longitudinal data analysis,^12^ and a review by the *Belgian Health Care Knowledge Centre*,^13^ in the landmark Nationwide Italian study, it has been suggested to be of benefit and associated with a 90% reduction in sudden cardiac death in the 20-year period following its implementation.^14^ Therefore, despite the overall risk of sudden cardiac death ranges from 1 in 53 703 to 1 in 164 000 athlete-years,^15–17^ it is important to ensure that the athlete is fully aware of the risks of competitive sports participation, since the consequences are so devastating.

Box 1 **Example of challenging (‘grey zone’) cardiac conditions for eligibility decisions Ψ**

Presence of non-specific T-wave changes with a structurally normal heart

Reason: Highly prevalent and not necessarily associated with an adverse prognosis

Patients presenting with some, but not all, of the required criteria for the diagnosis of cardiomyopathy or channelopathy

Reason: Diagnosis of condition not possible and therefore risk still uncertain

Genotype-positive individuals who have not yet developed a heart disease phenotype

*Reason:* Certain conditions such as hypertrophic cardiomyopathy or long QT mutations may have incomplete penetrance, that is, individuals with a mutation will not necessarily develop the cardiac condition

Non-compacted ventricular cardiomyopathy in patients with no detectable signs of arrhythmic vulnerability

*Reason*: Highly prevalent condition with sparse data regarding sports

Brugada syndrome with no previous documentation of arrhythmic events

*Reason:* Events are more likely to occur at night during sleep rather than exercise

Primary prevention ICD recipients without clear association of ventricular dysrhythmia with exertion in the past and no previous history of ICD therapies, namely if not involved in sports with high risk of collision or lead damage

*Reason*: Preliminary data regarding the effectiveness and durability of ICDs in athletes during short-term/medium-term follow-up are now available.

Permanent pacemaker recipients, mainly if not involved in sports with high risk of collision or lead damage, and if not pacing-dependent

*Reason*: Risk of lead failure is unlikely and even if it occurs, patients will not be exposed to an immediate life-threatening situation

Ψ Note: This list does not aim to be exhaustive or cover all possible clinical scenarios. It is based on current guidelines/consensus and on the authors' personal experience and views. We believe that in some of these situations, or other cases of high complexity, a Joint Case Conference by a group of experts may be the best option to provide a well-informed and non-biased medical judgement. Also, the decision to consider these individuals eligible should rely on the availability of automatic external defibrillators and presence of trained staff for cardiopulmonary resuscitation.

ICD, implantable cardioverter defibrillator.

### Involved parties

#### The patient

The *General Medical Council* provides clear guidance in consenting patients and in the decision-making process.^5^ Individuals with mental capacity to decide for themselves should be allowed to play the main role in the decision-making process, as discussed on point 5 of the document. This should happen irrespectively of the risk of adverse events, whether it is very low (annual risk deemed to be <1%), uncertain or high, as long as athletes can only harm themselves (ie, if they are not placing other individuals at risk) with the decision of not abandoning their sporting career. In situations of a clearly life-threatening condition (some examples are presented in box 2),^10^ ^11^ the physician should play a safeguarding role as he/she has a duty of care, but the ultimate decision left to the individual as long as they are competent.^5^

Box 2 **Example of situations associated with very high risk for competition Ψ**

Risk of harm to other individuals: athletes or spectators

(ie, auto-racing, motorcycling or riflery)

Risk of direct and life-threatening harm resulting from loss of consciousness in the evidence of a very high probability of arrhythmic events during competition

(ie, fall from great height, drowning, heavy body or cranial trauma)

Documentation of exercise/catecholaminergic driven life-threatening sustained ventricular arrhythmias in patients already on maximal β blocker dose, for example, CPVT, long QT syndrome

Documentation of high degree or third-degree atrioventricular block, proven not to be caused by hypervagotonic states, and causing symptoms, in patients without a permanent pacemaker

Patients with severe and non-reversible pulmonary hypertension in the presence or absence of congenital heart disease

Obstructive hypertrophic cardiomyopathy with severe dynamic gradient requiring appropriate intervention

Fixed obstructive lesions, for example, aortic stenosis, subaortic membrane

High risk of aortic rupture, for example, ascending aortic aneurysms in Marfan syndrome

Athletes previously requiring defibrillation during competition, namely if concerns exist regarding possible refractoriness or resistance of the arrhythmia and the availability of defibrillators

Ψ Note: This list does not aim to be exhaustive or cover all possible clinical scenarios. It is based on current guidelines/consensus and on the author's personal experience and views.

CPVT, cathecolaminergic polymorphic ventricular tachycardia.

Confirmation that an athlete understands the risks involved and has the necessary maturity to exercise autonomy and responsibility for their own health is critical and may require separate expert assessment if this is in doubt or contested. In some countries, the question of how much liberty an athlete has and the maximum level of risk they may be allowed to assume when making this decision, or which situations can be classified as too high risk, is still open to debate. Understanding the natural history of the disease, the probability and rate of progression, the risk of fatal events and the severity of resulting harm with sporting participation, as well as whether reasonable treatment interventions or taking specific precautions would sufficiently reduce or even eliminate risk of injury must constitute key components.^6^

#### The role of the physician

The task of the physician in this scenario is complex and should not be restricted to a final decision or judgement regarding disqualification from sporting competition. We suggest the main aim of the physician should be counselling the athlete on their condition, its likely natural history under different circumstances (ie, involvement in sporting participation or not), potential treatments or methods to potentially reduce risk, highlighting gaps in knowledge in medical practice pertaining to sport and their condition and protecting an athlete's health in all its dimensions (not only physical, but also psychological).

The *General Medical Council Good Medical Practice Document* states that doctors should ‘work in partnership with patients. Listen to, and respond to, their concerns and preferences. Give patients the information they want or need in a way they can understand. Respect patients’ right to reach decisions with you about their treatment and care’.^18^

A clear diagnosis of the medical problem is critical, and the physician should decide which method of screening is more appropriate, be it history-taking and physical examination, 12-lead resting ECG or echocardiogram for all patients, based on his/her perceived risk, and views on the sensitivity and cost-efficacy of the screening process. However, obtaining a conclusive diagnosis may sometimes present challenges, as some disease entities may be incompletely understood (eg, the thin line between normal physiological states and disease, eg, isolated non-compaction of the left ventricle^19^), or inconclusive (cases of non-specific repolarisation abnormalities with normal echocardiographic and MRI findings), or sometimes multiple disease states can overlap such that teasing out individual risks can prove challenging (box 3).^10^ ^11^ ^20^ Such complex decisions are best made by a group of experts in a Joint Case Conference or Multi-Disciplinary Team Meeting fully documenting the patient's clinical condition, investigations and consensus of risk of sudden death, prognosis and long-term management.

Box 3 **Causes and examples of situations that can deceive screening (false negatives) Ψ**

- (A) Intermittent phenotype
  Brugada syndrome
  Long QT syndrome
- (B) Phenotype only develops following exposure to an aggression
  Viral myocarditis
  Commotio cordis
- (C) Insidious onset and unpredictable development of phenotype
  Hypertrophic cardiomyopathy
  Arrhythmogenic right ventricular cardiomyopathy
  Familial dilated cardiomyopathy

ECG changes precede the development of the typical echocardiographic phenotype. Therefore, a diagnosis may not yet be possible while screening is performed and presence of electrical criteria for left ventricular hypertrophy and negative T waves in a patient with no family history of cardiomyopathy may be classified as unspecific or athlete's heart if changes in the echocardiogram or MRI are non-diagnostic. However, progression to hypertrophic cardiomyopathy may be detected at a later stage if regular echocardiographic monitoring is performed. Normal screening tests can also be observed in patients with who later progress to develop a full phenotype.

Ψ—This list does not aim to be exhaustive or cover all possible clinical scenarios. It is based on current guidelines/consensus and on the author's personal experience and views.

It is of the utmost importance the physician clearly informs patients (and relatives if the patient is legally a minor) of their condition, the possible risks and seriousness of the situation, areas of evidence and knowledge gaps, which treatment options are available and their effectiveness, and all according to existing data or lack thereof. The clinician should highlight when these are not 100% predictive and if existing data are preliminary.^21^ ^22^

The question of how much information a physician must give to a patient may be contentious. The *General Medical Council* document on *Good Medical Practice* advises that the doctor must tell patients if treatment might result in a serious adverse outcome, even if the risk is very small, and physicians should also tell patients about less serious complications if they occur frequently (point 32).^18^ Not informing the patient about a risk the doctor thinks is negligible, and therefore assuming the patient shares the same opinion, can be perceived as medical paternalism. The concept of material risk is of importance in this topic.^23^ This is defined as ‘either a risk to which a reasonable person in the patient's position would be likely to attach significance or a risk that a doctor knows—or should reasonably know—would probably be deemed of significance by this particular patient’*.*^24^ Therefore, if information is likely to be of significance to the athlete, doctors should disclose it, and should not wait for the athlete to ask for it.

According to the *General Medical Council* guidance, doctors should respect athlete's decision even if it seems ‘wrong or irrational’ (point 43), and ascertain the decision is voluntary (point 41) and not happening as a result of pressure from a third party.^5^ This certainly will be alarming to many physicians when a patient is perceived to be at very high risk of sudden death (see examples of box 2).^10^ ^11^ In such circumstances, every endeavour should be taken to ensure that the athlete understands the risk and is protected accordingly. However, ultimately the decision lies in the hands of the athletes unless they are officially banned by the club or sports organisation. In spite of this, every effort should be made to create a close partnership with the physician, as avoiding screening or any form of medical contact can result in harm to the athlete.

It is important that the physician explains to athletes and their sports team/coaches from start that in some ‘grey zone’ situations with non-specific findings or when a final diagnosis is unclear, the potential risk of adverse outcomes or the lack of knowledge of risk in these situations. This also applies when all screening tests prove normal, or there is thought to be only a minor, but non-negligible risk. As no situation can be totally risk-free, it is important to explain there is still a lot we do not know about sudden cardiac death in young adults, and we must acknowledge that 21st century medicine is not, and is unlikely to ever be, 100% reliable in identifying all individuals at risk of sudden arrhythmic death.

The physician should liaise with the patient and sports team or organisation providing information regarding the probabilities of adverse events that can occur in the particular athlete, and need and frequency of future follow-up. Accordingly, support should be given in the choice of appropriate medical equipment and implementation of staff training (cardiopulmonary resuscitation, use of automatic external defibrillators, etc). Formal, regular assessment of the appropriate functioning of this system, equipment and staff training to detect and treat life-threatening situations should be made before the athlete returns to the field.

#### Responsibilities of the employer/sports team/organisation

**Figure 1:**
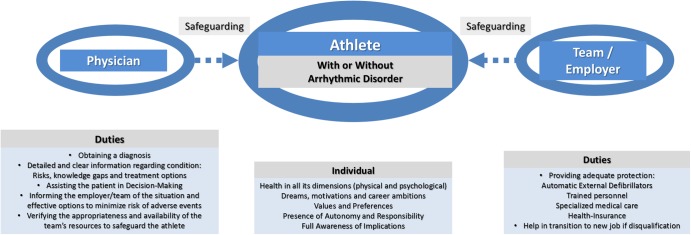
Aspects of the joint informed consent: roles and duties of the three involved parties.

The team or organisation has a duty of protecting the health and safety of participating athletes. In the UK, organisations have been deemed guilty of breach of duty to provide appropriate and urgent healthcare to athletes arising from complications occurring their sports activity. However, in some countries, uncertainty may exist regarding the legal duty of these organisations if patients voluntarily place themselves at increased risk in the context of an arrhythmic disorder.^6^ It is critical to ensure there is no evidence of coercion from the sports team/family, be it financial, competitive or otherwise. In the event of a joint informed consent between the athlete, physician and sports team, where each of the parties involved assumes its duties and obligations, we suggest the responsibilities of the team in offering adequate protection to the individual should include: (1) provision of automatic external defibrillators during training and competition; (2) training and availability of personnel for cardiopulmonary resuscitation and use of automatic external defibrillators; (3) provision of specialised medical care for cardiology (or other relevant) assessments for diagnosis, risk assessment, disease monitoring and necessary therapeutic interventions; (4) provision of adequate insurance for health-related complications resulting from competition; (5) for professional athletes agreeing on conditions for transition to a new occupation/role in the event of retirement due to health-related problems or events (figure 1). The latter may be of value to prevent situations of athletes being ‘forced’ to remain in competition because of concerns regarding their livelihood. However, there must be limits to this support, and the degree of involvement of the team in the transitioning process still requires clarification.

The right of a team physician, team consultants and an institution to restrict an athlete from participation is a very complex matter which is not discussed in this paper. Patient empowerment is a concept now being widely used in several fields of medicine,^25^ namely in the setting of diagnostic tests and therapeutic interventions. However, empowering patients through a shared-decision or an informed-decision model, or disempowering them through the use of a paternalist approach, should not preclude obtaining the athlete's consent.

Finally, a special note for a difficult situation meriting reflection: the athlete declining screening. There are situations where individuals with full capacity, and assuming that they have no known cardiac conditions, and/or are fully aware of possible risks, may refuse to undergo screening. Such instances should be analysed on a case-by-case basis, and the outcome will depend on each club's and sports league/competition policy or regulations. If the athlete's decision goes against regulations, the athlete will have no option, as screening will be mandatory in order to be eligible. However, if the regulation allows it and if the athlete is willing and aware of the involved risks, it is the authors' belief that the athlete should be empowered to make such a decision. However, necessary precautions (with AEDs and trained staff in CPR) should be made available during training and competition.

### Ultimate goal

We believe the widespread use of a ‘joint informed consent’ concept is a crucial step for making the decision process towards eligibility or disqualification in sport safer and more transparent. We believe this will lead to a better understanding of the potential risks involved in certain conditions, the appropriateness of competition, the need for lifelong monitoring and the best way to prevent a fatal outcome. The importance of a written document to outline the process, clarify the perceived current arrhythmic risk, the possibility of disease progression even in the face of a structurally normal heart and need for continuous monitoring should not be understated (see online supplementary material—appendix A). Also, the joint consent may be an effective way of ensuring clear and effective communication between the three involved parties (athlete, physician and sports team). It should be made clear that such a document can neither be perfect or inclusive of all potential outcomes but would act as a record of the best attempt to provide a comprehensive assessment of an individual athlete's current status in terms of risk of participating in competitive sport (see online supplementary material—appendix).

10.1136/openhrt-2016-000516.supp1 supplementary material

Ultimately, this will hopefully avoid denial of life-threatening heart conditions, forging closer relationships between the athlete and specialised health services and possibly improving the availability of care and chances of survival if a serious event occurs during competition. Awareness of potentially life-threatening situations (even if the risk is very low) may lead to wider availability of automatic external defibrillators on playing fields, and this would in turn be advantageous to athletes (with known or unknown cardiac conditions) and non-athlete bystanders/supporters.
