# Supplementary material for: Call for joint informed consent in athletes with inherited cardiac conditions
Source: Open Heart. 2017 Jan 30;4(1):e000516. doi: 10.1136/openhrt-2016-000516 (PMC5566987; doi:10.1136/openhrt-2016-000516)
Supplement: supplementary material [file openhrt-2016-000516supp001.pdf]

Supplementary Material

Annex A. Examples of Special Situations or Cases where a Joint Informed Consent can be of benefit

1. Waivers preventing future legal claims in the case of an adverse event

Steve Larkin, a baseball player with hypertrophic cardiomyopathy aged 18 was allowed to play for University of Texas after he and his parents signed a waiver that released the school from liability if he was injured or died as a result of the medical problem [1].

Cuttino Mobley, a former *National Basket Association* (NBA) basketball player with hypertrophic cardiomyopathy was allowed to play from 1999 to 2008 subject to the signing of a liability waver [2].

However, the legal enforceability of such waivers in the United States of America is unclear [3].

2. Disclosure of Material Risks

Not disclosing material risk to a patient with diabetes who gave birth to a baby, who as a result of complications during the delivery was born with severe disabilities, has recently resulted in conviction of a physician [4]. We believe the same principle may apply to athletes, as they should be entitled to avoid exposing themselves to the risk of a serious arrhythmic event, which they could otherwise have avoided, or, at least,

be allowed to decide whether or not to incur that risk. The American scientific statement recommends that physicians should fully inform athletes about possible material risks associated with competitive sport practice, preferably in writing [5].

3. Importance of Good Communication

The case of *Mr Radwan Hamed* is an example which illustrates this situation. This young *Tottenham Hotspur Football Club* soccer player in the UK, then aged 17, collapsed in his first official match in August 2006. An ambulance with a defibrillator arrived 15 minutes later and, although he survived, he developed permanent brain damage. His previous evaluation revealed the presence of T-wave abnormalities on ECG, but obvious features of hypertrophic cardiomyopathy were absent on a cardiac MRI performed one year earlier, and therefore "athlete's heart" was thought to be responsible for the ECG changes by the Cardiologist advising the football club. Nevertheless, in face of these ECG changes, closer monitoring, with annual review with echocardiogram and ECG, was advised by the Cardiologist, who suggested this was arranged by the club and he did not review the patient again. Unfortunately, clinical review, repeat ECG and echocardiography never took place, and training as a professional footballer resumed. A lawsuit followed and resulted in prosecution of: (1) the sports team for not arranging for the Claimant to be clinically reviewed or have follow-up and (2) the Cardiologist for not making it clear to the club that it was still a mandated requirement that a clinical review by a Cardiologist should be performed. Further consultation should have included a discussion between the patient, his family and the Cardiologist to clarify that the exact cause of the ECG

changes was unclear and there was a probably risk of serious arrhythmic events, albeit minor, thus allowing them to make a more informed decision [6].

4. Duties of Sports Clubs and Other Organizations

Michael Watson sustained life-changing neurologic damage during a fight for the *World Boxing Organization Super-Middleweight*. The fight was stopped in the final round when *Watson* became unable to defend himself. This happened as a result of subdural haematoma, and the boxer sustained left-sided paralysis and other physical and mental disability. The athlete brought an action against the *Board* as he claimed that it *"had been under a duty of care to see that all reasonable steps were taken to ensure that he received immediate and effective medical attention and treatment should he sustain injury in the fight".* The *Board* was found to be in breach, as the doctors who were present at the scene did not have the expertise in providing the resuscitation he required, nor did they have the right equipment, and this seemed to have contributed to his neurological damage [7]. We believe that this principle also applies to patient at risk of sustaining life-threatening events in the setting of arrhythmic heart disorders.

The new *American Heart Association / American College of Cardiology* scientific statement provides strong support to the availability of automatic external defibrillators in sports, whether competition, training, practice, either in schools or other organizations hosting athletic events, or providing training facilities for organized competitive athletic programs. Emphasis is also put on the availability of

basic life support by coaches and athletic trainers, and activation of emergency medical systems (all with Class I recommendations and Level of evidence B) [8].

5. Areas of Uncertainty

The following constitute three example of areas of uncertainty and serve to illustrate that sometimes physicians are not able to provide full reassurance to athletes regarding their current cardiac condition or risk: i. the use of automatic external defibrillators [9] and implantable cardioverter defibrillators [10], as although evidence seems to suggest safety and efficacy with their use, data and experience in sports practice is still scarce, and situations of automatic external defibrillator unresponsive arrhythmias have been previously reported. *Hank Gathers* was a *National Collegiate Athletic Association Division* basketball player who collapsed on a basketball court, and despite the availability of an automatic external defibrillator and ventricular fibrillation being recorded, efforts at resuscitation were unsuccessful. Although uncertainty exists regarding diagnosis, evidence suggests it was probably myocarditis [11]; ii. HCM patients deemed to be of very low risk as assessed through the new risk prediction model (HCM Risk-SCD) [12], as no risk stratification strategy will ever be able to predict malignant ventricular arrhythmias with absolute certainty and this risk prediction model has not been validated for athletes [12]; iii. Even though it has been associated with an increased risk of sudden cardiac death, non-compacted ventricular cardiomyopathy seems to be highly prevalent in athletes [13], but very sparse data is available regarding its safety in sports [14, 15].

Annex B. Particular Aspects of the Joint Informed Consent Process

1. Role of Joint Case Conferences or Multi-Disciplinary Team Meetings

A consensus approach reduces the exposure of an individual clinician to the risk of bias/lack of expertise in decision-making and helps to ensure that a balanced evidence-based assessment is made utilising shared expertise in the best interests of the patient. It is also likely that experience of working in a group such as this will help develop the decision-making processes in time, aiding all the physicians or healthcare practitioners involved.

2. Prevention of Disease Progression and Frequency of Monitoring

Preventative measures to limit disease progression and frequency of serial testing, even when in the presence of an apparently normal phenotypes (as some times disease may present only a few months or years after the initial evaluation), must be part of the discussion and may vary from case to case (Table 3).

3. Physician's Communication Skills

Physicians specialising in this area must develop good communication skills particularly pertinent to the framework of this discussion and information must be conveyed clearly, with simple language, and with an assessment of the

understanding of the situation by the patient, allowing him or her the necessary amount of time to discuss and ask questions. This knowledge will enable the athlete to participate properly in the decision-making process.

4. The Informed Consent Document

The three parties (athlete, treating physician and the team/organization) should be involved in the process of the joint informed consent. We believe that this would mark the conclusion of an initial decision-making process towards eligibility or disqualification, binding the parties together through an official document stating their individual obligations and roles, and acknowledging the potential risks associated with participating in sport and their acceptance of potential outcomes.

We recommend all parties should be involved in the formulation of a written document, which records the consensus (or potentially lack thereof) achieved after careful consideration of an individual's risks and benefits of participating in competition. As an alternative, the physician could also prepare a report comprising all information and recommendations given to the patient and sports team/organization, which should be accepted by the athlete and made available to the sports team. Even though it is still uncertain if in the United States of America this type of document could legally bind any of the parties involved, and thus physicians can still be vulnerable to lawsuits, it would act as a clear record of their decisions at that time point & serve as a transparent record for the physician, patient & parties involved [5].

5. Legal Value of the Concept

Even though the *"joint informed consent"* concept has not yet been accepted as a model for best practice, we feel it is important this issue is addressed and explored in the future. It is not considered an option or mandatory requirement in current guidelines [14, 15], but its future inclusion could be valuable. Judicial support or precedence for such an informed consent is still unknown and should be explored by national sports organizations but, in our opinion, the lack of legal clarity does not devalue this process from a medical perspective and should be pursued.

References

1. Larkin allowed to play for UT. The Victoria Advocate – Apr 25, 1992.

2. Mobley v Madison Square Garden LP, 2013 US Dist LEXIS (SD New York 2012).

3. Mitten MJ, Maron BJ. Legal considerations that affect medical eligibility for competitive athletes with cardiovascular abnormalities and acceptance of Bethesda Conference Recommendations. *J Am Coll Cardiol.* 1994;24(4):861-3.

4. Montgomery (Appellant) v Lanarkshire Health Board (Respondent) (Scotland) [2015] UKSC 11.

5. Mitten MJ, Zipes DP, Maron BJ, Bryant WJ. Eligibility and Disqualification Recommendations for Competitive Athletes With Cardiovascular Abnormalities: Task Force 15: Legal Aspects of Medical Eligibility and Disqualification Recommendations: A Scientific Statement From the American Heart Association and American College of Cardiology. J Am Coll Cardiol. 2015;66(21):2447-50.

6. Hamed v Mills & Tottenham Hotspur Football Club [2015] EWHC 298 (QB). Royal Courte of Justice, Strand, London, WC2A 2LL, Date: 16/02/15, available online: http://www.39essex.com/content/wp-content/uploads/2015/02/Final-Hamed-v-Mills.pdf , last accessed 1st August 2015

7. Michael Alexander Watson v British Boxing Board of Control Limited, World Boxing Organisation Incorporated [2000] EWCA Civ 2116

8. Zipes DP, Link MS, Ackerman MJ, Kovacs RJ, Myerburg RJ, Estes NA 3rd. Eligibility and Disqualification Recommendations for Competitive Athletes With Cardiovascular Abnormalities: Task Force 9: Arrhythmias and Conduction Defects: A Scientific Statement From the American Heart Association and American College of Cardiology. J Am Coll Cardiol. 2015;66(21):2412-23.

9. Drezner JA, Toresdahl BG, Rao AL, Huszti E, Harmon KG. Outcomes from sudden cardiac arrest in US high schools: a 2-year prospective study from the National Registry for AED Use in Sports. *Br J Sports Med.* 2013;47(18):1179-83.

10. Lampert R, Olshansky B, Heidbuchel H, Lawless C, Saarel E, Ackerman M, Calkins H, Estes NAM, Link MS, Maron BJ, Marcus F, Scheinman M, Wilkoff BL, Zipes DP, Berul CI, Cheng A, Law I, Loomis M, Barth C, Brandt C, Dziura J, Li F, Cannom D. Safety of Sports for Athletes With Implantable

Cardioverter-Defibrillators: Results of a Prospective, Multinational Registry. *Circulation.* 2013;127:2021-2030.

11. Maron BJ. Sudden death in young athletes: lessons from the Hank Gathers affair. N *Engl J Med.* 1993;329:55–7.

12. O'Mahony C, Jichi F, Pavlou M, Monserrat L, Anastasakis A, Rapezzi C, Biagini E, Gimeno JR, Limongelli G, McKenna WJ, Omar RZ, Elliot PM, Hypertrophic Cardiomyopathy Outcomes Investigators. A novel clinical risk prediction model for sudden cardiac death in hypertrophic cardiomyopathy (HCM risk-SCD). Eur Heart J. 2014;35:2010-20.

13. Gati S, Chandra N, Bennett RL, Reed M, Kervio G, Panoulas VF, Ghani S, Sheikh N, Zaidi A, Wilson M, Papadakis M, Carré F, Sharma S. Increased left ventricular trabeculation in highly trained athletes: do we need more stringent criteria for the diagnosis of left ventricular non-compaction in athletes? *Heart.* 2013;99(6):401-8.

14. Maron BJ, Zipes DP, Kovacs RJ; American Heart Association Electrocardiography and Arrhythmias Committee of the Council on Clinical Cardiology, Council on Cardiovascular Disease in the Young, Council on Cardiovascular and Stroke Nursing, Council on Functional Genomics and Translational Biology, and the American College of Cardiology. Eligibility and Disqualification Recommendations for Competitive Athletes With Cardiovascular Abnormalities: Preamble, Principles, and General Considerations: A Scientific Statement From the American Heart Association and American College of Cardiology. Circulation. 2015;132(22):e256-61.

15. Pelliccia A, Fagard R, Bjørnstad HH, et al. Recommendations for competitive sports participation in athletes with cardiovascular disease. A consensus document from the Study Group of Sports Cardiology of the Working Group of Cardiac Rehabilitation and Exercise Physiology, and the Working Group of Myocardial and Pericardial diseases of the European Society of Cardiology. *Eur Heart J*. 2005;26:1422– 45.
